# Nanofibrous Formulation of Cyclodextrin Stabilized Lipases for Efficient Pancreatin Replacement Therapies

**DOI:** 10.3390/pharmaceutics13070972

**Published:** 2021-06-27

**Authors:** Gergő Dániel Tóth, Adrienn Kazsoki, Benjámin Gyarmati, András Szilágyi, Gábor Vasvári, Gábor Katona, Lajos Szente, Romána Zelkó, László Poppe, Diána Balogh-Weiser, György T. Balogh

**Affiliations:** 1 Department of Physical Chemistry and Materials Science, Budapest University of Technology and Economics, Műegyetem rkp. 3, H-1111 Budapest, Hungary; tothgergodaniel@edu.bme.hu (G.D.T.); gyarmati.benjamin@vbk.bme.hu (B.G.); szilagyi.andras@vbk.bme.hu (A.S.); 2 University Pharmacy Department of Pharmacy Administration, Semmelweis University, Hőgyes Endre utca 7-9, H-1092 Budapest, Hungary; kazsoki.adrienn@pharma.semmelweis-univ.hu (A.K.); zelko.romana@pharma.semmelweis-univ.hu (R.Z.); 3 Department of Pharmaceutical Technology, Faculty of Pharmacy, University of Debrecen, Nagyerdei u. 98, H-4032 Debrecen, Hungary; vasvari.gabor@pharm.unideb.hu; 4 Faculty of Pharmacy, Institute of Pharmaceutical Technology and Regulatory Affairs, University of Szeged, Eötvös u. 6, H-6720 Szeged, Hungary; katona.gabor@szte.hu; 5 Cyclolab Cyclodextrin Research & Development Laboratory Ltd., Illatos út 7, H-1097 Budapest, Hungary; szente@cyclolab.hu; 6 Department of Organic Chemistry and Technology, Budapest University of Technology and Economics, Műegyetem rkp. 3, H-1111 Budapest, Hungary; poppe.laszlo@vbk.bme.hu; 7 Department of Chemical and Environmental Process Engineering, Budapest University of Technology and Economics, Műegyetem rkp. 3, H-1111 Budapest, Hungary; 8 Institute of Pharmacodynamics and Biopharmacy, Faculty of Pharmacy, University of Szeged, Eötvös u. 6, H-6720 Szeged, Hungary

**Keywords:** pancreatic enzyme replacement therapy (PERT), lipase, cyclodextrin, electrospinning, nanofibrous enzyme formulation

## Abstract

Enzyme replacement therapies (ERT) have been of great help over the past 30 years in the treatment of various lysosomal storage disorders, including chronic pancreatitis and its common complication, exocrine pancreatic insufficiency. Research shows that difficulties in designing such drugs can be overcome by using appropriate additives and various enzyme immobilization techniques. Cyclodextrins (CDs) can be considered as a promising additive for enzyme replacement therapies, as they are known to enhance the activity of enzymes in a complex process due to their specific binding. In this study, we investigated the formulation of lipases (from *Aspergillus oryzae* and *Burkholderia cepacia*) paired with different cyclodextrins in poly(vinyl alcohol) (PVA) nanofibers by electrospinning technique. We examined the effect of the presence of cyclodextrins and nanoformulation on the lipase activity. The rheological and morphological characterizations of precursors and nanofibers were also performed using a viscometer as well as electron and Raman microscope. We found that by selecting the appropriate CD:lipase ratio, the activity of the investigated enzyme could be multiplied, and cyclodextrins can support the homogeneous dispersion of lipases inside the solid formula. In addition, the entrapment of lipases in PVA nanofibers led to a significant increase in activity compared to the preformulated precursor. In this way, the nanofibrous formulation of lipases combining CDs as additives can provide an efficient and sustainable possibility for designing novel solid medicines in ERT.

## 1. Introduction

### 1.1. Enzyme Replacement Therapies (ERT)

Lysosomal storage disorders (LSDs) are inherited metabolic diseases that cause an atypical build-up of various toxic materials in the body’s cells, resulting in enzyme deficiencies. [1]. Enzyme replacement therapy (ERT), or in other words, the idea of replacing a deficient enzyme, first emerged in the 1960s in connection with the treatment of LSDs, when de Duve and Brandy found that people with LSDs who retained some residual enzyme activity expressed less severe symptoms in contrast to patients with complete enzyme deficiencies [2,3,4]. In 1991 the first ERT was approved by the FDA to treat Gaucher disease [5]. Since then, the ERT methods proved to be widely successful in treating various LSDs, such as Fabry disease, lysosomal acid lipase deficiency, Hurler syndrome, Hunter syndrome, and Maroteaux–Lamy syndrome [6,7]. While these methods are really effective at treating the symptoms of LSDs, they do not provide a solution for the inherent problem of insufficient enzyme production [8]. The biggest disadvantage of using ERTs, is that their effectiveness depends deeply on whether the ERT can be targeted to the disease-affected tissues. This often requires modifications to the enzyme, as purified enzymes are often not targeted to the lysosomes. In addition, certain tissues express low biocompatibility to enzymes (proteins) [9]. Moreover, treating neurological symptoms poses a great challenge because ERTs that are given intravenously are unable to cross the blood–brain barrier. Common workarounds to this problem include using higher ERT doses, modifying the enzymes to facilitate their movement through the blood–brain barrier, and providing the ERT in the form of intrathecal injections. Furthermore, the annual cost of ERTs is rather high. Depending on the patient, the use of exogeneous enzymes may also cause unwanted immune reactions [10,11]. Enzymes are capable of catalyzing specific reactions under physiological conditions, and in general they express low toxicity. These attributes would make them promising candidates as drugs; however, there are several drawbacks to using them [12]. Some enzymes are hard to acquire in pure, nontoxic forms, which makes them highly expensive to use [13]. Another problem is that enzymes administered to patients can go through rapid biodegradation due to enzymatic proteolysis [14]. The administered protein can cause immunological reactions, due to being considered as “foreign” to the patient’s body [15]. Finally, guaranteeing that the administered enzyme is delivered to the appropriate specific site can be a great challenge. Many of these problems can be solved by immobilizing the enzymes [16]. Microcapsules, enzymes immobilized in artificial cells [17], cross-linked enzyme aggregates [18], spherical polymer beads [19], and single enzyme nanoparticles [20] are the most common forms.

### 1.2. Pancreatic Enzyme Replacement Therapy (PERT)

Pancreatic enzymes perform the lead role in the hydrolytic breakdown of macronutrients into smaller metabolites [21]. Chronic pancreatitis (CP) is a progressive fibro-inflammatory disorder that leads to the long-term destruction of ductal, acinar, and islet cells. The most common complication of this disease (>50%) is exocrine pancreatic insufficiency (EPI), which causes inadequate pancreatic secretion of digestive enzymes, resulting in the reduced metabolism of nutrients [22]. EPI is known to reduce absorption of essential fatty acids and thus lipophilic vitamins, such as A, D, E, and K and various other compounds [23]. After secretion, the intraluminal enzyme activity gradually decreases during the transit through the intestines, but the rate of inactivation differs significantly from enzyme to enzyme. Amylase and protease enzymes retain a significant portion of their initial activity, but lipases get inactivated rapidly due to the lack of triglycerides. This process can be slowed down in the presence of lipase substrates [24,25]. During CP, lipases go through fast inactivation, and extra pancreatic enzymes are unable to compensate the lost digestive enzymatic activity. To make up for deficiency, it is necessary to deliver the required amount of enzyme to the duodenal lumen together with the food consumed. Although we know how much enzyme activity a regular human needs for an average meal, if the patient is given unprotected “bare” enzymes, the desired therapeutic effect is likely not achieved due to rapid inactivation. For this reason, it is often inevitable to use more than ten times the theoretically required amount of enzyme to achieve sufficient results. A common solution to this problem is to formulate the enzyme to be administered into an acid-resistant coated tablet or capsule. However, this is significantly complicated by the physiology of the gastric emptying process. The stomach does not excrete inert particles larger than 2 mm with food, which means that the enzymes are unable to trace the nutrients into the small intestine [26,27]. The most effective modern pancreatin formulations are delivered to patients in the form of acid-resistant, pH-sensitive microspheres. These are excreted from the stomach together with the gastric pulp into the duodenum, where they release their enzyme content as the pH increases [28]. It is important to note that because the release of the active ingredient from these formulations is not instantaneous, the delay thus introduced may shift the maximum site of absorption along the length of the digestive tract [29].

### 1.3. Lipases in PERT

The most used lipases in PERT are human and porcine pancreatic lipases. The problem with human lipases is that they get easily deactivated by proteases; therefore, for preparations containing this kind of lipase, it is advisable to use a reduced amount of protease to ensure the survival of the lipases [30]. Efficacy can be further enhanced by the use of various fungal enzymes (e.g., lipase from *Rhizopus arrhizus*, *Aspergillus niger*, *Rhizopus oryzae*, and *Aspergillus oryzae*) [31,32,33] and bacterial enzymes (e.g., lipase from *Burkholderia cepacia* and *Burkholderia plantarii*) [34,35]. Fungal enzymes are known to be significantly less stable than bacterial ones [36]. Accordingly, studies have shown that these enzymes rapidly lose a significant portion of their activity in the presence of bile acid [37]. Research has found that the lipolytic activity of bacterial lipases is maintained to a greater extent in the gastrointestinal tract than porcine pancreatic lipase, and so the use of these types of enzymes can significantly reduce the amount of lipase required to treat steatorrhea [38].

### 1.4. Cyclodextrins as Additives

Cyclodextrins (CDs) are cyclic oligosaccharides composed of chair-conformed d-glucopyranose units linked by α (1-4) glycosidic bonds. Due to the conformation of their monomers, CDs have a characteristic truncated cone-shaped structure. The outer surface of these oligosaccharide funnels is typically polar and hydrophilic, while the interior (the so-called cavity) is formed by the hydrophobic carbon backbone of the monomeric D-glucopyranose units. Due to this special molecular structure and amphiphilic behavior (interior hydrophobic cavity region and exterior hydrophilic rim), CDs are able to form water-soluble inclusion complexes with poorly water-soluble organic molecules [39]. Because of their saccharide nature, CDs, like linear dextrins, are not toxic to humans [40]. Thus, CDs are a preferred choice by the pharmaceutical industry for improving the water solubility of the active ingredients in various pharmaceutical formulations. Examples of drugs containing CDs include intravenous Caverject^®^ Dual (an α-CD containing drug, meant to treat erectile dysfunction), the β-CD containing antiallergic Cetirizine tablet, and the anti-inflammatory Voltaren Ophtha^®^ eye drops containing hydroxypropyl γ-CD. Although CDs are able to enhance the biocatalytic properties of enzymes and their use can increase the effectiveness of different formulations, their application requires precise planning because either under- or overdosing CDs can deteriorate the efficacy of the preparation. One of the major limitations of using enzymes as biocatalysts is that they are only able to express a fraction of their activity in non-natural media. Implementing such transformations in a heterogeneous phase reaction can overcome solubility issues, but such diffusion-controlled processes are inherently slower than those in a homogeneous phase. By using CDs, the apparent water solubility of the substrate can be increased, thereby allowing for an efficient biocatalytic conversion of poorly water-soluble organic molecules [41]. CDs can also mitigate the inhibitory effect of the substrate, or the product in certain enzymatic reactions, by the complexation of the interfering component, thus keeping its concentration low [42]. Due to their steric effects, CDs are able to bind prochiral molecules in their cavity in such a way that the enzymatic attack can only reach them from a prominent enantioselective side, thus greatly increasing the enantioselectivity of the enzyme [43].

**Figure 1:**
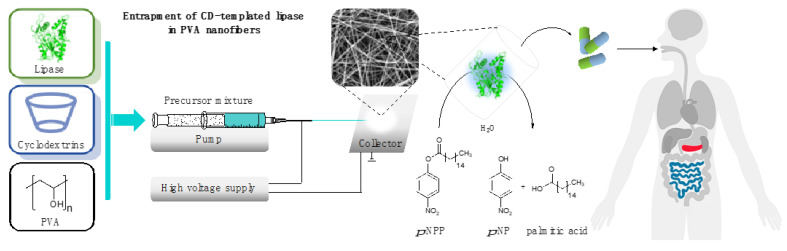
Formulation of cyclodextrin-templated lipase by entrapping in poly(vinyl alcohol) (PVA) nanofibers, applying electrospinning technique as a potential active pharmaceutical ingredients for pancreatin replacement therapy.

In this study we investigate the effect of selected cyclodextrins on the enzymatic activity of lipases from two different strains (*Burkholderia cepacia* and *Aspergillus oryzae*), which are accepted enzymes in PERT. Among nanoformulation methods, electrostatic fiber formation is applied, as it enables great variability regarding the polymers, provides mild conditions for sensitive proteins, and is cost-effective and scalable, making it easy to integrate it in different stages of pharmaceutical development. Although the entrapment of lipases in poly(vinyl alcohol) (PVA) nanofibers by electrospinning technique has already been performed [44,45], the effect of cyclodextrins during the nanoformulation of lipases has not been reported yet. In this study, the rheology of precursors containing lipases and CDs as well as the morphology of the nanofibers created from these precursors is examined. The presence and distribution of lipases in the fibers is studied by Raman mapping. The enzymatic function of lipases is examined in the hydrolysis of *p*-nitrophenyl palmitate (*p*-NPP) as a natural and widely accepted transformation to explore the lipase specific activity. The enzyme activity is determined under standard conditions optimized for hydrolytic lipase activity in a medium that simulates the environment of human intestine (Figure 1).

## 2. Materials and Methods

### 2.1. Materials

The *p*-nitrophenyl palmitate (*p*-NPP), *p*-nitrophenol (*p*-NP), Mowiol^®^ 18-88 (poly(vinyl alcohol), PVA; M_W_ ≈ 130 kDa), sodium chloride, tris(hydroxymethyl)aminomethane (Tris), Triton X100, gum arabic, sodium taurocholate, glacial acetic acid, sodium hydroxide pellets, *Burkholderia cepacia* lipase (PS Lip), and *Aspergillus oryzae* lipase (AO Lip) were purchased from Sigma-Aldrich (Saint Louis, MO, USA). β-Cyclodextrin (B-CD), 2-hydroxypropyl-β-cyclodextrin (HPB-CD, DS = 4.7), 2-hydroxypropyl-γ-cyclodextrin (HPG-CD, DS = 6.3), randomly methylated β-cyclodextrin (RAMEB-CD), and sulfobutylated-β-Cyclodextrin (SBB-CD, DS = 6.8) were a kind gift from Cyclolab Ltd. (Budapest, Hungary). In all cases, the water was purified by a Millipore Milli Q water purification system (Bradford, MA, USA). In addition, 2-propanol (IPA) was purchased from Merck (Darmstadt, Germany); the Kreon^®^ 25000 (Lipase:Amylase:Protease = 25,000:18,000:1000 Unit) gastric resistance hard capsule was a product of Mylan EPD Ltd. (Budapest, Hungary), and the Pangrol^®^ 25000 (Lipase:Amylase:Protease = 25,000:22,500:1250 Unit) gastric resistance hard capsule was produced by Berlin-Chemie Menarini (Berlin, Germany).

### 2.2. Standard Lipase Activity Assay

A standard lipase activity assay was performed according to the widely used and accepted method by Kordel [46]. To the Tris buffer (900 μL, pH = 8.0, 50 mM, 0.4% (*w*/*v*) Triton X100, 0.1% (*w*/*v*) gum arabic), a solution of *p*-NPP (100 μL, 16.5 mM, dissolved in 2-propanol) was added. The resulting solution was homogenized by vigorous shaking on an orbital shaker at 850 rpm using Heidolph TitraMax 1000 (Schwabach, Germany), and 150 μL of lipase solution (0.1 mg × mL^−1^ lipase, containing CD in 1:0, 1:1, 1:3, or 1:6 lipase:CD mass ratio, in Tris buffer (pH = 8.0, 50 mM, 0.4% (*w*/*v*) Triton X100, 0.1% (*w*/*v*) gum arabic)) was added to the resulting mixture to initiate the test reaction. The reaction mixture was placed on an orbital shaker (at 450 rpm) at 37.0 °C. After 5, 15, 30, and 60 min, 60 μL of the reaction mixture was sampled and diluted with 940 μL of Tris buffer (pH 8.0, 50 mM) in a semi-micro PMMA cuvette (BRAND^®^, Sigma-Aldrich, Saint Louis, MO, USA). Then, the *p*-NP absorbance values were determined by a UV–VIS spectrophotometer (Genesys-2, Thermo Fisher Scientific Inc., Waltham, MA, USA) at the specific wavelength of *p*-NP (λ = 400 nm, see ESI in Figure S1a). All experiments were performed in triplicate. The effect of CDs on *p*-NPP hydrolysis under the conditions of a standard assay without lipases was also investigated (see ESI in Figure S2a).

### 2.3. Lipase Activity Assay in Fed-State Simulated Intestinal Fluid (FeSSIF) System

To prepare a “blank FeSSIF” solution, 1.187 g of sodium chloride, 0.865 g of glacial acetic acid, and 100.0 mL of water were added to 0.404 g of sodium hydroxide pellets, and the pH of the resulting solution was adjusted to 5.0. Then, 0.4125 g of sodium taurocholate was dissolved in 500.0 mL of blank FeSSIF solution, followed by adding a solution of 86.6 mg of *p*-NPP, 0.50 g of Triton X100, and 0.125 g of gum arabic in 2-propanol (1.477 mL), and the resulting mixture was sonicated for 60 min by applying 45 kHz in a Sonorex Digitec DT 255 ultrasonic bath (Bandelin Electronic GmbH, Berlin, Germany). To 1.0 mL of FeSSIF solution, we added 150 μL of lipase solution (0.1 mg × mL^−1^ lipase, dissolved in blank FeSSIF solution, containing CD in 1:0, 1:1, 1:3 or 1:6 lipase:CD mass ratio), and the resulting mixture was placed on an orbital shaker (at 450 rpm) at 37.0 °C. After 5, 15, 30, and 60 min, 40 μL of the reaction mixture was sampled, diluted with 960 μL of blank FeSSIF solution in a semi-micro PMMA cuvette, and the *p*-NP absorbance values were determined by the UV–VIS spectrophotometer at the specific wavelength of *p*-NP λ = 318 nm (see ESI in Figure S1b). Every experiment was performed in triplicate. The effect of CDs on *p*-NPP hydrolysis was also investigated under conditions consistent with an assay in FeSSIF without the presence of lipases (see ESI in Figure S2b).

### 2.4. Determination of Lipase Activity and Conversion of p-NPP Hydrolysis

The concentration of *p*-NP was calculated using its extinction coefficient (ε), which was determined for the conditions of the standard assay and an assay in FeSSIF using calibration curves (ε = 14,733 M^−1^ × cm^−1^ for standard, ε = 8686 M^−1^ × cm^−1^ for FeSSIF assay). In order to compare the efficacy of the different lipase-catalyzed hydrolyses, the values of conversion (*c*, %), specific enzyme activity (*U*_E_, U × g^−1^), and specific enzymatic activity for formulations (*U*_F_, U × g^−1^) were determined on the basis of the measured absorbance data (AU) by the following equations:

*c* = *n*_P_ × (*n*_S_ + *n*_P_)^−1^ × 100 (1)

*U*_E_ = *n*_P_ × (*t* × *m*_E_)^−1^ (2)

*U*_F_ = *n*_P_ × (*t* × *m*_F_)^−1^ (3)

where *n*_P_ (in μmol) and *n*_S_ (in μmol) are the molar amounts of product (P) and substrate (S), respectively; *t* is the time (in min); *m*_E_ is the mass of the enzyme (in g); and *m*_F_ is the mass (in g) of the formula (lipase entrapped in nanofibers or commercially available medicinal product).

### 2.5. Rheological Analysis

The viscosity of the CD-free and CD and lipase-containing precursor PVA mixtures used for fiber formation was determined using a Physica MCR 301 rheometer (Anton Paar, Graz, Austria). A probe with a cone–plate geometry (with a diameter of 25 mm and cone angle of 1°) was used for the tests. The measuring chamber of the instrument was thermostated at 25.0 °C for each sample, and the interval of shear rate was 1–631 s^−1^. Every experiment was performed in triplicate.

### 2.6. Entrapment of CD-Templated Lipases in Poly(vinyl alcohol) Nanofibers

The PVA solution (10% *w*/*w*, aqueous solution) required for the preparation of precursors used for fiber formation was obtained by dissolving solid Mowiol^®^ 18-88 granules in deionized water. Dissolution was performed in a 50 °C water bath using a magnetic stirrer (IKA RH basic, IKA GmbH, Staufen im Breisgau, Germany). To prepare the precursors, native lipase and CD (in 1:1, 1:3, or 1:6 lipase:CD mass ratio) were dissolved in 2.00 g of PVA solution in an amount such that the enzyme would make up 10% of the weight of the fiber produced from the solution. The precursors were homogenized by vigorous shaking by vortex for 3 min (IKA Mixer Vortex Shaker MS 2, IKA GmbH, Staufen im Breisgau, Germany). The precursors were introduced airtight into a single use sterile syringe (3 mL Omnifix Luer Lock, B Braun, Melsungen, Germany), from which they were then transferred to a 22 G diameter emitter (except for PS Lip HPB-CD samples, where a 18 G emitter was used) by a syringe pump through a PTFE tube (1/16 OD, 250 μL dead volume). For electrospinning experiments, a laboratory electrospinning machine was applied (SpinCube Laboratory Electrospinning system, SpinSplit LLC, Budapest, Hungary). For the electrostatic fiber formation processes, a collector–emitter distance of 10 cm, a feed rate of 0.08 μL s^−1^, and a voltage of 11.3–13.3 kV were used. During the spinning processes, the room temperature was 23 °C, and the relative humidity was 26% under continuous air condition. The nanofibrous product was collected on a thin layer of aluminum foil attached to the collector surface. The nanofibrous products were dried for 1 h at room temperature and then stored at 4 °C.

### 2.7. Morphological Analysis

Morphology of nanofiber matrices was studied by a JSM JEOL-5500LV (JEOL, Tokyo, Japan) SEM-EDS scanning electron microscope (SEM), and samples were placed on a copper grid. To ensure adequate surface conductivity of the samples, the tested samples were coated with gold in a few atomic layers of thickness by a nebulizer (Polaron sc760 mini sputter coater, Thermo VG Microtech, Waltham, MA, USA) using Ar-plasma, at 10 mA for 180 s. The images were taken in the high vacuum mode of the SEM. SEM images were processed using digital image evaluation software (ImageJ 1.52r, U.S. National Institutes of Health) to determine the average diameter and standard deviation of the nanofibers (n = 100 measurement points).

### 2.8. Raman Mapping Analysis of Nanofibrous Products

Raman mapping of nanofibers was performed using a Thermo Fisher DXR dispersive Raman instrument (Thermo Fisher Scientific Inc., Waltham, MA, USA) equipped with a CCD camera and a diode laser operating at a wavelength of 780 nm. For sample preparation, a glass slide was covered with aluminum foil containing electrospun nanofibers. A Raman chemical map was obtained from a 100 × 100 μm surface of different nanofibers with a 1 × 1 μm spectral resolution while applying a laser power of 12 mW at a 50 µm slit aperture size. The spectrum of the chemical map was recorded with an exposure time of 2 s and acquisition time of 6 s, for a total of 32 scans per spectrum in the spectral range 3300–200 cm^−1^ with cosmic ray and fluorescence corrections. Each Raman map was normalized in order to eliminate the intensity deviation between the measured areas.

### 2.9. Investigation of Nanofibrous Lipase and Commercially Available Formulas

To test the activity of the nanofiber formulations, 5.0 mg of each formulation was dissolved in 5.0 mL of the appropriate buffer according to standard or FeSSIF assay (see Section 2.2 and Section 2.3). Dissolution was almost instantaneous. The resulting solutions were homogenized by vigorous shaking using an orbital shaker (at 450 rpm). Subsequently, the procedures were performed as described in Section 2.2 and Section 2.3, respectively, by initiating the reactions with 150 μL of the solutions obtained by dissolving the fibrous products.

To prepare the “preformulated precursor” mixture used to determine the effects of nanofiber formation on the activity enhancing effect of CDs, solid Mowiol^®^ 18-88 granules, lipase enzyme (AO Lip or PS Lip), and CD (in the case of PS Lip: B-CD, HPB-CD in 1:3 lipase:CD mass ratio; in the case of AO Lip: HPB-CD, SBB-CD in 1:1 lipase:CD mass ratio) were weighed to form a powder mixture that contains 10% (*w*/*w*) lipase. The resulting mixture was dissolved in the appropriate buffer according to standard assay or an assay in FeSSIF (see Section 2.2 and Section 2.3) to form a 1 mg ml^−1^ solution. The resulting solutions were homogenized by vigorous shaking using an orbital shaker (at 450 rpm). Subsequently, the procedures were performed as described in Section 2.2 and Section 2.3, respectively, by initiating the reactions with 150 μL of the solutions obtained by dissolving the powder mixtures.

To test the activity of commercial drugs, one capsule was broken from each formulation; 5.0 mg of the load was dissolved in 5.0 mL of the appropriate buffer according to standard or FeSSIF assay (see Section 2.2 and Section 2.3), and the resulting mixtures were homogenized for 1 h using an orbital shaker (at 450 rpm). Subsequently, the procedures were performed as described in Section 2.2 and Section 2.3, respectively, by initiating the reactions with 150 μL of the solutions obtained by dissolving the commercial drugs. An analysis of the samples taken from the reactions was performed as described in Section 2.4; every experiment was performed in triplicate.

## 3. Results and Discussion

### 3.1. Effect of Cyclodextrins on the Native Lipase Activity

**Figure 2:**
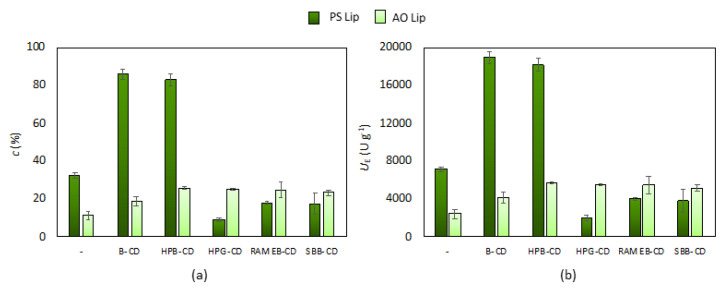
Effect of cyclodextrins (B-CD: β-cyclodextrin, HPB-CD: 2-hydroxypropyl-β-cyclodextrin, HPG-CD: 2-hydroxypropyl-γ-cyclodextrin, RAMEB-CD: randomly methylated β-cyclodextrin, SBB-CD: sulfobutylated-β-cyclodextrin) on (**a**) the conversion (c, %) of *p*-NPP hydrolysis catalyzed by lipase from *Burkholderia cepacia* (PS Lip, in dark green) and from *Aspergillus oryzae* (AO Lip, in light green) and on (**b**) the specific enzyme activity (U_E_, U × g^−1^) after 5 min at 37.0 °C in standard lipase assay (according to Section 2.2). Cyclodextrin:lipase ratios were 1:3 (*w*/*w*) for PS Lip and 1:1 (*w*/*w*) for AO Lip.

We first examined the effect of the presence of different CDs (B-CD, HPB-CD, HPG-CD, RAMEB-CD, and SBB-CD) on two lipases from different strains (*Burkholderia cepacia* lipase and *Aspergillus oryzae*) applied for the lipase catalyzed hydrolysis of *p*-NPP. The reaction mixture was prepared as described in Section 2.2, in which both selected lipases were tested in the presence of all selected CDs and in a CD-free reaction environment. For each lipase–CD pair, three different lipase:CD mass ratios (1:1, 1:3, 1:6) were examined in 1 h interval (see ESI in Figures S2 and S3). The results confirmed that under- or overdosing CDs mitigated the catalytic efficiency, but the optimal amount of CD can significantly enhance it. Of the weight ratios tested, 1:1 for AO Lip and 1:3 for PS Lip were the most effective for the hydrolysis of fatty acid ester (Figure 2). From the data presented, it is clear that in the case of PS Lip, most of the CDs tested reduced the activity of the enzyme even at the adequate 1:3 lipase:CD mass ratio, but the presence of B-CD as well as HPB-CD brought significant improvement in lipase function. The pairing of B-CD or HPB-CD with PS Lip resulted in more than a twofold conversion achievable as compared to the native lipase within 1 h reaction time. In the case of AO Lip at the appropriate lipase:CD mass ratio (1:1), all CDs improved the enzyme function. The best results were obtained in the presence of HPB-CD and SBB-CD, as illustrated by the high conversions and low standard deviation of the summarized data collected from all the experiments performed after 5 min (Figure 2) and longer periods (15, 30, and 60 min of reaction time; see ESI in Figure S3). During further research, the activity of PS Lip:B-CD (1:3), PS Lip:HPB-CD (1:3), AO Lip:HPBCD (1:1), and AO Lip:SBB-CD (1:1) was investigated.

After determining the most beneficial lipase:CD mass ratio and the best CD additives to both tested enzymes, we studied the enzyme activity in FeSSIF assay as described in Section 2.3 to mimic the conditions of intestine. Our results indicate that for PS Lip, the presence of both B-CD and HPB-CD led to a similarly large increase in activity as in the standard assay (Table 1), and conversion is growing continuously within the investigated 1 h period (see ESI in Figure S4a). The remarkably positive effect of B-CD and HPB-CD on the activity of PS Lip could be caused by specific electrostatic interactions and the hydrogen bond formation between the hydroxyl group of CDs and some amino acid side chains of the lipase. This beneficial property of β-cyclodextrins has been reported in studies about lipase from *Candida rugosa* [47,48]. A light increase in enzyme activity was also observed for AO Lip, although to a much lesser extent than for PS Lip (Table 1). Notably, the conversions that were achieved in the reactions catalyzed by AO Lip in the FeSSIF assay after 5 min reached a constant value (see ESI in Figure S4b). It can be assumed that due to the acidic environment of FeSSIF, the fungal AO Lip was deactivated more rapidly than the bacterial PS Lip. Thus, the increase in conversion observed after 1 h of reaction likely occurred only in the first 5–10 min of the process. If this is the case, the presence of CD could partially protect AO Lip even under the unpleasant conditions until the point of total activity loss.

The effect of the five selected CDs at the corresponding amount to 1:1 and 1:3 lipase:CD mass ratios in *p*-NPP hydrolysis was investigated under the conditions of a standard assay and an assay in FeSSIF as well for 1 h. Results showed, that CDs without lipases have no remarkable effect on *p*-NP formation (see ESI in Figure S2).

### 3.2. Solid Formulation of Lipases by Entrapment in Poly(vinyl alcohol) Nanofibers

Electrostatic fiber formation (electrospinning) is an easy-to-perform, fast, scalable, low-excipient process with the ability to turn enzymes into a solid formula that is easy to handle, to store, and to distribute into defined doses in drugs. Our previous studies showed that poly(vinyl alcohol) nanofibers were well applicable for lipase immobilization [46,49,50,51]. The specific enzyme activity of entrapped lipases could be increased due to the improved dispersion of enzyme molecules within the nanofibers with high specific surface area. Moreover, some substrate-like additives could further improve the activity of the lipases [46,49,50,51]. To study the solid formulation of lipase–CD pairs selected from the previous electrospinning experiments, we performed the nanofiber formation as described earlier (see Section 2.6). Since the viscosity of the precursor is a key parameter during electrospinning, the rheology of the precursors was examined (see Section 2.5). It was shown that the copresence of CDs and lipases did not really affect the viscosity of the precursors from a technological point of view (Table 2). The morphology of electrospun formulations was also investigated by scanning electron microscopy (SEM, described in Section 2.7). An analysis of the average fiber diameter indicates that the CDs and lipases increased the thickness of the fibers compared to the CD and enzyme-free PVA nanofibers.

**Figure 3:**
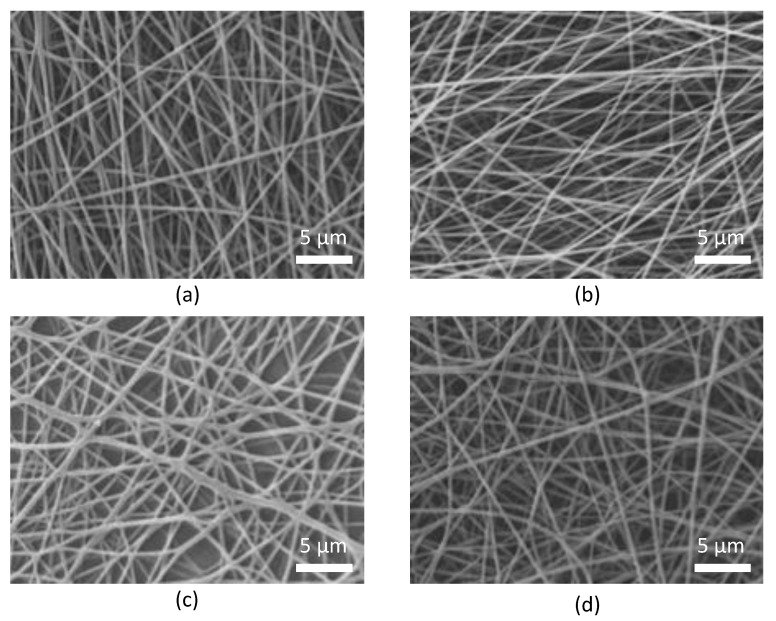
Scanning electron microscopic (SEM) images of lipase *Burkholderia cepacia* (PS Lip) entrapped in poly(vinyl alcohol) (PVA 18-88) nanofibers with (**a**) β-cyclodextrin (B-CD) and (**b**) with 2-hydroxypropyl-β-cyclodextrin (HPB-CD); and lipase from *Aspergillus oryzae* (AO Lip) entrapped in PVA nanofibers with (**c**) HPB-CD and with (**d**) sulfobutylated-β-cyclodextrin (SBB-CD) at magnification 3500×. All the fibers presented on the SEM images contain 10% (*w*/*w*, relative to the sum mass of the fibers) lipase and CD in specific lipase:CD mass ratios (PS Lip:CD = 1:1, AO Lip:CD = 1:3 (*w*/*w*)).

The SEM images presented in Figure 3 show that the morphology of the obtained fibers is uniform and that it indeed falls in the submicron size range. All the tested lipase–CD pairs proved to be suitable for formulation by nanofiber formation. In all cases, the good nanofiber-forming nature of the compositions resulted in continuous and stable fiber formation with constant parameters of the electrospinning process.

**Figure 4:**
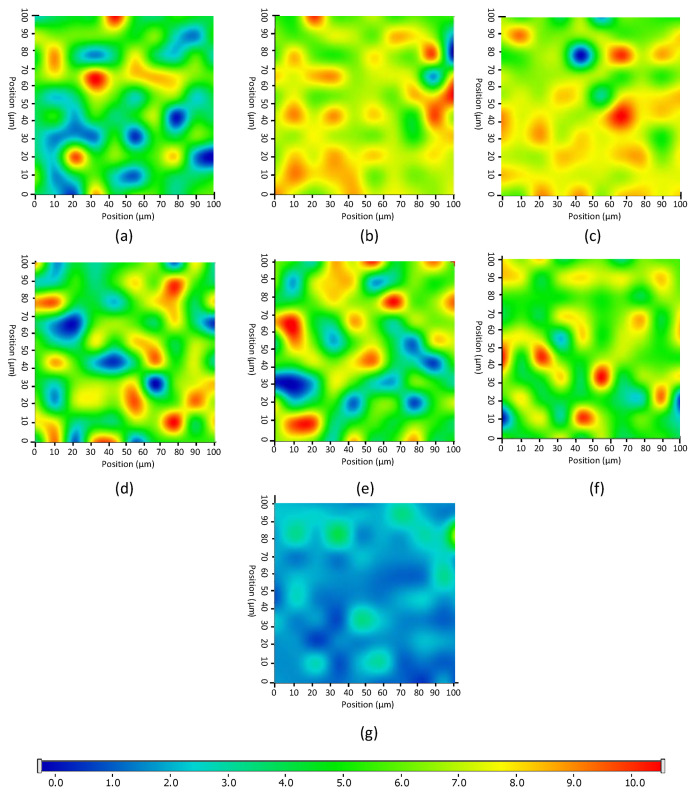
Raman chemical maps of different poly(vinyl alcohol) (PVA)-based electrospun nonwoven tissues indicating the distribution of lipases with relative occurrence: (**a**) AO Lip entrapped in PVA nanofibers, (**b**) AO Lip entrapped in PVA nanofibers with HPB-CD, (**c**) AO Lip entrapped in PVA nanofibers with SBB-CD, (**d**) PS Lip entrapped in PVA nanofibers, (**e**) PS Lip entrapped in PVA nanofibers with B-CD, (**f**) PS Lip entrapped in PVA nanofibers with HPB-CD, and (**g**) neat PVA-based nanofibrous material. BCD: β-cyclodextrin, HPB-CD: 2-hydroxypropyl-β-cyclodextrin, SBB-CD: sulfobutylated-β-cyclodextrin.

As Raman spectroscopy is sensitive to the secondary protein structure, it is a suitable tool form investigating the distribution of lipase in the nanofibers [52]. To ensure a smooth and even surface in order to eliminate focusing errors of samples during analysis, the sample holder glass slide was covered with a slice of aluminum foil containing electrospun nanofibers and placed under the Raman microscope. A further advantage of the application aluminum foil was the low spectral background and lack of spectral features, which may interfere with the measurement. The vibrational bands of carbonyl stretching of amide I region (1600–1700 cm^−1^) show strong Raman intensity, which corresponds to the secondary structure of protein. In the Raman spectrum (Figure S5), the characteristic amide I band arises principally from the C–O stretching vibration of the peptide group of lipases. The α-helix (1663 cm^−1^) and β-strands (1702 cm^−1^) can be clearly observed especially in case of PS Lip [53]. The sharp bands in both spectra in the range 1420–1508 cm^−1^ indicates C–H bending in the protein structure. The amide III region of enzymes can be also observed at 1100–1147 cm^−1^, corresponding to N–H bending, C–N stretching of peptide bonds. These spectral characteristics differ from other applied components; therefore, Raman mapping can be suitable for further analysis. After the initial structural characterization of the two lipases, the distribution of enzymes in the different nanofibers was investigated by Raman mapping (the corresponding microscopic images of the samples are shown in ESI, in Figure S6). For the localization of proteins, the Raman spectra of the non-entrapped lipases were used as reference, whose frequency of occurrence is shown in the chemical maps, which represents statistical distribution of the specific chemical entities (Figure 4). The different colors of the chemical map indicate the relative intensity change of lipase specific spectral components in the nanofibers. The red areas indicate its strong existence, and the green areas show a mixed composition, whereas the blue areas mark the regions of the map where the spectral resolution contains different spectra that are characteristic for another components. This can be also clearly seen in the case of pure enzyme and CD-free PVA fiber (Figure 4g), which was selected as control for the measurement. The results revealed that the distribution of enzymes is dispersed more evenly in the presence of CDs, as shown by the remarkably high relative intensity values (in red) of the Raman map, whereas in the case of CD-free compositions, the lipase can be found in well-defined packages, forming less uniform structure inside the PVA fiber.

After the selected formulations were found to be suitable for electrostatic fiber formation, the activity of the lipase–CD pairs immobilized in PVA nanofibers was examined similarly as for the nonformulated native lipases. The data in Table 3 show that the conversion values obtained for lipases entrapped without CDs in both the standard assay and the FeSSIF assay are consistent with the results shown in Figure 2 and Table 1. The nanofibrous lipase formulas dissolved immediately and completely in both buffered systems (standard assay and assay in FeSSIF), and thus the release of lipases was momentary in all case. However, a significantly smaller increase in activity was observed for lipases immobilized in the presence of CDs than for non-immobilized enzymes-CD pairs. This can be rationalized by assuming that the interaction of CD with the dissolved PVA in the assay changes the lipase:CD weight ratio, which was shown to have a notable effect on enzyme activity. This may also be the explanation in the case of PS Lip where the presence of HPB-CD in the nanofibers resulted in a much more remarkable deterioration in activity compared to B-CD. Due to the hydroxypropyl groups, HPB-CD is likely able to form a stronger interaction with PVA, thus upsetting the sensitive lipase:CD weight ratio even more. The same trend can be observed for AO Lip as well, which was true for the investigated 1 h reaction time (see ESI in Figure S7). Although the fall of activity for AO Lip was less significant than for PS Lip, the presence of both CDs caused a deterioration in enzyme activity, with HPB-CD causing larger changes, as in the case of PS Lip, whose effect is almost constant during the examined 1 h reaction period (see ESI in Figure S6). The smaller rate of deterioration in activity can likely be explained by the early inactivation mentioned (see Section 3.1).

**Figure 5:**
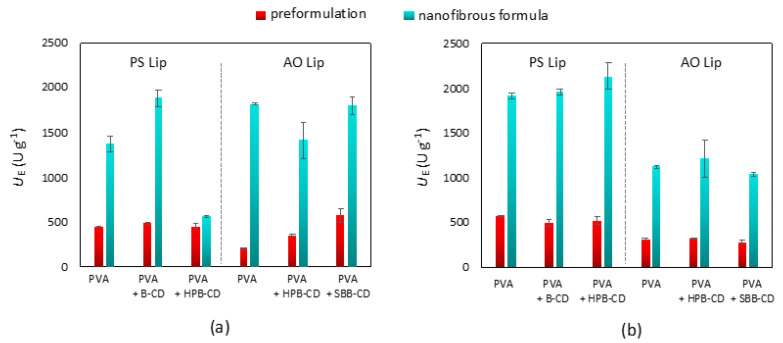
Comparison of the specific enzyme activity (*U*_E_, U × g^−1^) of preformulated precursor (in red) and nanofibrous formulations (in aqua) of lipases from *Burkholderia cepacia* (PS Lip) and from *Aspergillus oryzae* (AO Lip) templated with different cyclodextrins in *p*-NPP hydrolysis after 1 h at 37.0 °C in (**a**) standard assay and (**b**) assay in FeSSIF (according to Section 2.8). To carry out the tests, 5 mg of the nanofibrous formulations that contain 10% (*w*/*w*, relative to the sum mass of the fibers) lipase and CD in the appropriate lipase:CD mass ratio (PS Lip:CD = 1:1, AO Lip:CD = 1:3 (*w*/*w*)) were dissolved in 5 mL of the appropriate buffer solution (Tris for standard assay and blank FeSSIF for the assay in FeSSIF). The same composition of solutions were prepared by dissolving Mowiol^®^ 18-88, lipase, and CD-s in the appropriate buffers (preformulated precursors); 150 μL of the resulting solutions were used to initiate the test reactions in 1.0 mL of standard assay and 1.0 mL of the assay in FeSSIF. B-CD: β-cyclodextrin, HPB-CD: 2-hydroxypropyl-β-cyclodextrin, SBB-CD: sulfobutylated-β-cyclodextrin.

Another possible explanation for the deterioration in the activity of lipase–CD pairs is that the protein or the additive used lose their effectiveness due to the changes suffered during the fiber formation process. This would mean that nanofiber formation is not suitable to perform the desired formulation. This issue was examined by comparing the activity of the preformulated precursors (see Section 2.9) with the activity of nanofiber formulations in both the standard assay and the assay in FeSSIF. The results depicted in Figure 5 clearly indicate that the activities of the dissolved nanofibrous lipase formulations far exceeded those obtained by applying the preformulated precursors in each case. In the case of the assay in FeSSIF, results using the AO Lip were in all cases inferior to those obtained with PS Lip. The kinetic curves shown in Figures S7 and S8 indicate that this phenomenon was caused by the previously mentioned early inactivation of the AO Lip. Thus, the increase in activity, which can be observed as an effect of fiber formation, is most likely caused by the tight arrangement of the CDs and proteins that occurs in the nanofibers. After the dissolution of fibers, CDs and lipases remain closer to each other than they would in the preformulated precursors, resulting in a much more prevalent activity-enhancing effect of the CDs.

### 3.3. Comparison of Lipase Activity of Cyclodextrin-Templated Nanofibers with Commercially Available Medicines

To compare the efficacy of our CD-templated lipases entrapped in PVA nanofibers, two commercially available medicines that include combined enzyme preparations for pancreatin replacement, i.e., Kreon^®^ 25000 and Pangrol^®^ 25000 (which are gastric resistance hard capsules), were selected because they are easier to investigate and handle than simple hard tablets. As expected, the pharmaceutical formulations (Kreon^®^ 25000 and Pangrol^®^ 25000) performed much more efficiently in the assay in FeSSIF (Figure 5b) than in the standard assay, since these formulations were designed to perform optimally in such an environment. The quite similar activities of the two drugs were also expected, since the nominal lipase activity of the two selected formulations is the same and the type of formulas are quite similar. Our results showed that the AO Lip plus CD formulations performed with comparable efficacy during the test reaction to the two drugs, while the mass specific activities of bacterial PS Lip-containing formulations (with the exception of PS Lip-HPBCD) significantly overperformed the ones of the commercial drugs. Investigation of the conversion values of *p*-NPP hydrolysis in the full period (1 h) showed that with nanofibrous lipases, the conversion was significantly higher than with hard capsules in general (Table 4). In the case of PS Lip templated with B-CD, AO Lip, and AO Lip templated with SBB-CD, almost total conversion could be observed in the standard assay. Remarkably, the nanofibrous formulation of lipases provide continuously growing product formation (see ESI in Figures S6 and S9), but the lipase powder from hard capsules approaches only a stationary value far from the total conversion (see ESI in Figure S10). The conditions for the assay in FeSSIF resulted in moderate conversion values, and the differences between the commercial medicines and nanofibrous formulas are not so tremendous as in the case of the standard assay. However, at least more than a twofold increase in the specific enzyme activity (*U*_E_) can be realized for every nanofibrous lipases as compared with the commercial formulations (Table 4). PS Lip with HPB-CD provided the highest conversion, which surpassed the results of Kreon^®^ or Pangrol^®^. This specific activity enhancement is of particular interest: considering the total weight of the granules filled in the hard-capsule drugs as lipases, a much lower lipase weight in the fibers exhibits significantly higher activity, being further enhanced in the presence of CDs. In the assay in FeSSIF, the formulated PS Lip and AO Lip expressed two and four times the activity of the tested drugs, respectively.

## 4. Conclusions

We found that the activity of the tested lipases (lipase from *Burkholderia cepacia* and from *Aspergillus oryzae*) was very sensitive to the quality and quantity of cyclodextrins (CDs) used as an additive in the lipase-catalyzed hydrolysis of *p*-nitrophenyl palmitate. Of the five investigated CDs compared (β-cyclodextrin, 2-hydroxypropyl-β-cyclodextrin, 2-hydroxypropyl-γ-cyclodextrin, randomly methylated β-cyclodextrin, sulfobutylated-β-cyclodextrin), the two that caused the most remarkable increase in activity for both selected lipases were studied in more detail, and then an optimal lipase:CD weight ratio was determined for both proteins, using the ratio with which the CDs significantly improved the catalytic activity of lipases. We also found that of the lipases tested, PS Lip showed significantly greater resistance to the more acidic environment of the simulated intestinal fluid. Our results showed that lipases with CDs can be successfully entrapped in poly(vinyl alcohol) nanofibers by solvent electrospinning, and that the morphology and the lipase distribution in the nanofibers seemed to be uniform. In the lipase–CD formulations created by electrostatic fiber formation, a much smaller activity-enhancing effect of CD was observed, which most likely indicates an interaction between the CDs and the material used as the carrier matrix. To achieve optimal synergy, it would be worthwhile to investigate in more detail the effect of the CD:lipase weight ratio immobilized in the nanofibers. The activity of the reported nanofibrous formula is comparable to the selected drugs, and in some cases this showed higher hydrolytic activity, based on which we can say that it is worthwhile to further study and optimize the production conditions of the nanofiber formulation.

Based on the obtained results, we can say that it is worthwhile to study the formulation of the selected lipases for pancreatic enzyme replacement therapy. In order for the formulation to become an effective enzyme-based product, cyclodextrins can be a promising additive that can positively influence the enzymatic activity. Thereafter, delivery of the composition to the appropriate intestinal tract should be accomplished. The most obvious formulation solution to this problem could be encapsulation in a solid material. The application of electrospinning technique for the entrapment of lipases in polymeric nanofibers can be a unique, rapid, fine-tunable and scalable method for the solid formulation of sensitive enzymes. Thus, nanofibrous enzyme entrapment can be a promising formulation tool, and by filling the fibers into capsules, the proper targeted absorption of the formulated enzymes can be ensured.

## Figures and Tables

**Table 1:** Effect of cyclodextrins on the conversion (*c*, %) in hydrolysis of *p*-NPP in FeSSIF catalyzed by lipase from *Burkholderia cepacia* (PS Lip) and from *Aspergillus oryzae* (AO Lip) and on the specific enzyme activity (*U*_E_, U × g^−1^).

Lipase	Cyclodextrin	*c* (%)	*U*_E_ (U × g^−1^)
PS Lip	-	18.2 ± 0.7	10,827 ± 429
	B-CD	78.1 ± 2.5	47,178 ± 125
	HPB-CD	79.3 ± 0.9	47,045 ± 537
AO Lip	-	12.0 ± 0.4	7120 ± 222
	HPB-CD	13.7 ± 0.2	8135 ± 111
	SBB-CD	12.5 ± 0.3	7400 ± 178

B-CD: β-cyclodextrin, HPB-CD: 2-hydroxypropyl-β-cyclodextrin, SBB-CD: sulfobutylated-β-cyclodextrin. After 5 min: to 1.0 mL of FeSSIF solution 150 μL of lipase solution was added, i.e., 0.1 mg × mL^−1^ lipase, dissolved in blank FeSSIF solution, containing CD in the appropriate lipase:CD mass ratio (PS Lip:CD = 1:1, AO Lip:CD = 1:3 (*w*/*w*)), and the resulting mixture was placed on an orbital shaker (450 rpm) at 37.0 °C.

**Table 2:** Effect of lipases from *Burkholderia cepacia* (PS Lip) and from *Aspergillus oryzae* (AO Lip), and cyclodextrins on the viscosity of precursor mixture and on the diameter of nanofibers.

Lipase	Cyclodextrin	Precursor Viscosity (mPas)	Fiber Diameter (nm)
-	-	426 ± 8	241 ± 48
PS Lip	-	356 ± 23	335 ± 51
	B-CD ^1^	446 ± 41	316 ± 49
	HPB-CD ^2^	479 ± 63	368 ± 48
AO Lip	-	354 ± 14	368 ± 67
	HPB-CD ^2^	399 ± 2	412 ± 117
	SBB-CD ^3^	384 ± 14	371 ± 76

^1^ β-cyclodextrin, ^2^ 2-hydroxypropyl-β-cyclodextrin, ^3^ sulfobutylated-β-cyclodextrin. To prepare the precursors, native lipase and CD (PS Lip:CD = 1:1, AO Lip:CD = 1:3 (*w*/*w*)) was dissolved in 2.00 g of Mowiol^®^ 18-88 PVA solution (10% (*w*/*w*), aqueous solution) in an amount such that the enzyme made up 10% (*w*/*w*) of the solids content in the solution. To prepare the precursors, native lipase and CD (PS Lip:CD = 1:1, AO Lip:CD = 1:3 (*w*/*w*)) was dissolved in 2.00 g of Mowiol^®^ 18-88 PVA solution (10% (*w*/*w*), aqueous solution) in an amount such that the enzyme made up 10% (*w*/*w*) of the solids content in the solution.

**Table 3:** Effect of cyclodextrins on the conversion (*c*, %) of *p*-NPP hydrolysis catalyzed by nanofibrous formulations of lipase from *Burkholderia cepacia* (PS Lip) and from *Aspergillus oryzae* (AO Lip), and on the specific enzyme activity (*U*_E_, U × g^−1^) after 5 min in standard lipase assay and assay in FeSSIF system.

Lipase	Cyclodextrin	Standard Assay ^1^		FeSSIF Assay ^2^	
		*c* (%)	*U*_E_ (U × g^−1^)	*c* (%)	*U*_E_ (U × g^−1^)
PS Lip	-	34.2 ± 1.3	7530 ± 296	17.6 ± 0.5	104,515 ± 309
	B-CD	55.7 ± 4.3	12,249 ± 957	15.7 ± 0.3	9297 ± 155
	HPB-CD	4.5 ± 0.1	983 ± 27	11.2 ± 0.4	6664 ± 234
AO Lip	-	22.4 ± 0.7	4921 ± 164	7.3 ± 0.4	4340 ± 255
	HPB-CD	18.4 ± 1.2	4048 ± 271	11.4 ± 0.2	6752 ± 117
	SBB-CD	21.6 ± 1.4	4760 ± 315	12.0 ± 0.6	7120 ± 331

^1^ To Tris buffer (900 μL, pH = 8.0, 50 mM, 0.4% (*w*/*v*) Triton X100, 0.1% (*w*/*v*) gum arabic) a solution of *p*-NPP (100 μL, 16.5 mM, dissolved in 2-propanol) was added. To initiate the test reactions 150 μL of lipase solution was added, i.e., 5 mg nanofibrous lipase formulation, containing 10% (*w*/*w*, relative to the sum mass of the fibers) lipase and CD in the appropriate lipase:CD mass ratio (PS Lip:CD = 1:1, AO Lip:CD = 1:3 (*w*/*w*)), dissolved in 5.0 mL blank Tris solution, and the resulting mixture was placed on an orbital shaker (450 rpm) at 37.0 °C. ^2^ To 1.0 mL of FeSSIF solution 150 μL of lipase solution was added {5 mg nanofibrous lipase formulation, containing 10% (*w*/*w*, relative to the sum mass of the fibers) lipase and CD in the appropriate lipase:CD mass ratio [PS Lip:CD = 1:1, AO Lip:CD = 1:3 (*w*/*w*)], dissolved in 5.0 mL blank FeSSIF solution} and the resulting mixture was placed on an orbital shaker (450 rpm) at 37.0 °C. B-CD: β-cyclodextrin, HPB-CD: 2-hydroxypropyl-β-cyclodextrin, SBB-CD: sulfobutylated-β-cyclodextrin.

**Table 4:** Comparison of conversion (*c*, %) and specific enzyme activity of the different lipase formulas: nanofibrous and commercially available medicinal products (*U*_F_, U × g^−1^), in hydrolysis of *p*-NPP after 1 h at 37 °C, in the standard assay and the assay in FeSSIF (according to Section 2.8).

Lipase	Cyclodextrin	Standard Assay		Assay in FeSSIF	
		*c* (%)	*U*_F_ (U × g^−1^)	*c* (%)	*U*_F_ (U × g^−1^)
**Nanofibrous formula**					
PS Lip	-	74.9 ± 4.8	137 ± 9	38.7 ± 0.5	191 ± 3
	B-CD	102.6 ± 4.7	188 ± 9	39.6 ± 0.5	196 ± 3
	HPB-CD	30.7 ± 0.5	56 ± 1	43.6 ± 3.5	216 ± 18
AO Lip	-	99.1 ± 0.8	182 ± 10	22.7 ± 0.3	112 ± 1
	HPB-CD	77.3 ± 11.1	142 ± 20	24.6 ± 4.1	122 ± 20
	SBB-CD	96.3 ± 7.0	177 ± 13	21.0 ± 0.4	104 ± 2
**Marketed pharmaceutical product**					
Kreon^®^ 25000		6.7 ± 1.3	12 ± 2	10.9 ± 0.1	54 ± 1
Pangrol^®^ 25000		3.4 ± 0.3	6 ± 1	10.8 ± 0.5	54 ± 3

^1^ To Tris buffer (900 μL, pH = 8.0, 50 mM, 0.4% (*w*/*v*) Triton X100, 0.1% (*w*/*v*) gum arabic) a solution of *p*-NPP (100 μL, 16.5 mM, dissolved in 2-propanol) was added. To initiate the test reactions 150 μL of lipase solution was added, i.e., 5 mg nanofibrous lipase formulation, containing 10% (*w*/*w*, relative to the sum mass of the fibers) lipase and CD in the appropriate lipase:CD mass ratio (PS Lip:CD = 1:1, AO Lip:CD = 1:3 (*w*/*w*)), dissolved in 5.0 mL blank FeSSIF solution, and the resulting mixture was placed on an orbital shaker (450 rpm) at 37.0 °C. ^2^ To 1.0 mL of FeSSIF solution 150 μL of lipase solution was added, i.e., 5 mg nanofibrous lipase formulation, containing 10% (*w*/*w*, relative to the sum mass of the fibers) lipase and CD in the appropriate lipase:CD mass ratio (PS Lip:CD = 1:1, AO Lip:CD = 1:3 (*w*/*w*)), dissolved in 5.0 mL blank FeSSIF solution, and the resulting mixture was placed on an orbital shaker (450 rpm) at 37.0 °C.

## Data Availability

Not applicable.

## Supplementary Materials

The following are available online at https://www.mdpi.com/article/10.3390/pharmaceutics13070972/s1, Figure S1: Absorption spectrum of p-nitrophenol (p-NP) at various concentration in (a) the media of standard lipase assay (see MS in Section 2.2) and (b) fed-state simulated intestinal fluid (FeSSIF, see MS in Section 2.3) at 37.0 °C, Figure S2: Investigation of the effect of CDs on the p-NPP hydrolysis without lipase in (a) standard assay and (b) assay in FeSSIF, with different CD amounts at 37.0 °C for 1 h, Figure S3: Effect of cyclodextrins (B-CD, HPB-CD, HPG-CD, RAMEB-CD, SBB-CD) on the p-NP formation from p-NPP hydrolysis catalyzed by PS Lip at 37.0 °C in standard lipase assay (according to Section 2.2 in MS) with the presence of different cyclodextrin:lipase ratio (a) 1:1, (b) 1:3 and (c) 1:6 (*w*/*w*), Figure S4: Effect of cyclodextrins on the p-NP formation from p-NPP hydrolysis catalyzed by lipase from AO Lip at 37.0 °C in standard lipase assay (according to Section 2.2 in MS) with the presence of different cyclodextrin:lipase ratio (a) 1:1, (b) 1:3 and (c) 1:6 (*w*/*w*), Figure S5: Effect of cyclodextrins (B-CD, HPB-CD, SBB-CD) on the p-NP formation from p-NPP hydrolysis catalyzed by (a) PS Lip and (b) AO Lip in assay in FeSSIF (according to Section 2.3 in MS), PS Lip:CD = 1:3, AO Lip:CD = 1:1 (*w*/*w*) at 37.0 °C, Figure S6: Raman spectra of native enzymes, PS Lip colored with black and AO Lip colored with red, Figure S7: Mosaics of different products indicating the investigated area during Raman mapping: (a) AO Lip-PVA, (b) AO Lip-HP-β-CD-PVA, (c) AO Lip-SB-β-CD-PVA, (d) PS Lip-PVA, (e) PS Lip-β-CD-PVA, (f) PS Lip-HP-β-CD-PVA and (g) initial PVA filament, Figure S8: Effect of cyclodextrins (B-CD, HPB-CD, (PS Lip:CD = 1:3, *w*/*w*)) on the p-NP formation from p-NPP hydrolysis catalyzed by PS Lip entrapped in PVA nanofibers (a) in standard assay and (b) assay in FeSSIF at 37.0 °C (according to Section 2.9 in MS), Figure S9: Effect of cyclodextrins (B-CD, HPB-CD, SBB-CD (PS Lip:CD = 1:3, AO Lip:CD = 1:1, *w*/*w*)) on the p-NP formation from p-NPP hydrolysis catalyzed by (a) PS Lip and (b) AO Lip in preformulation mixture containing PVA in standard assay at 37.0 oC (according to Section 2.9 in MS), Figure S10: Effect of cyclodextrins (B-CD, HPB-CD, SBB-CD (PS Lip:CD = 1:3, AO Lip:CD = 1:1, *w*/*w*) on the p-NP formation from p-NPP hydrolysis catalyzed by (a) PS Lip and (b) AO Lip in preformulation mixture containing PVA in FeSSIF assay at 37.0 °C (according to Section 2.9 in MS), Figure S11: Effect of cyclodextrins (HPB-CD, SBB-CD (AO Lip:CD = 1:1, *w*/*w*)) on the p-NP formation from p-NPP hydrolysis catalyzed by AO Lip entrapped in PVA nanofibers (a) in standard assay and (b) assay in FeSSIF at 37.0 °C (according to Section 2.9 in MS), Figure S12: Investigation of commercially available medicines, Pangrol® and Kreon® by the formation of p-NP from p-NPP hydrolysis in (a) standard assay and (b) assay in FeSSIF (according to Section 2.9 in MS).
