# Supplementary material for: Nanofibrous Formulation of Cyclodextrin Stabilized Lipases for Efficient Pancreatin Replacement Therapies
Source: Pharmaceutics. 2021 Jun 27;13(7):972. doi: 10.3390/pharmaceutics13070972 (PMC8308945; doi:10.3390/pharmaceutics13070972)
Supplement: Supplementary file 1 [file pharmaceutics-13-00972-s001.zip › pharmaceutics-1259255-supplementary.pdf]

pharmaceutics

MDPI

Supplementary Materials: Nanofibrous Formulation of Cyclodextrin Stabilized Lipases for Efficient Pancreatin Replacement Therapies

Gergő Dániel Tóth, Adrienn Kazsoki, Benjámin Gyarmati, András Szilágyi, Gábor Vasvári, Gábor Katona, Lajos Szente, Romána Zelkó, László Poppe, Diána Balogh-Weiser and György T. Balogh

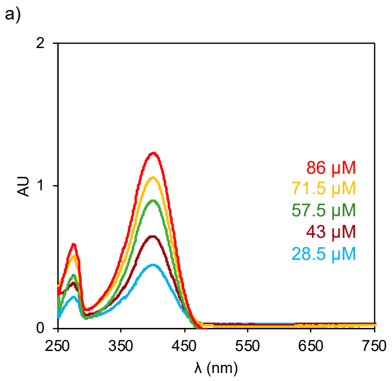

Figure S1. Absorption spectrum of *p*-nitrophenol (*p*-NP) at various concentration in **a**) the media of standard lipase assay (see in MS Section 2.2) and **b**) Fed stated simulated intestinal fluid (FeSSIF, see in MS Section 2.3) at 37.0 °C.

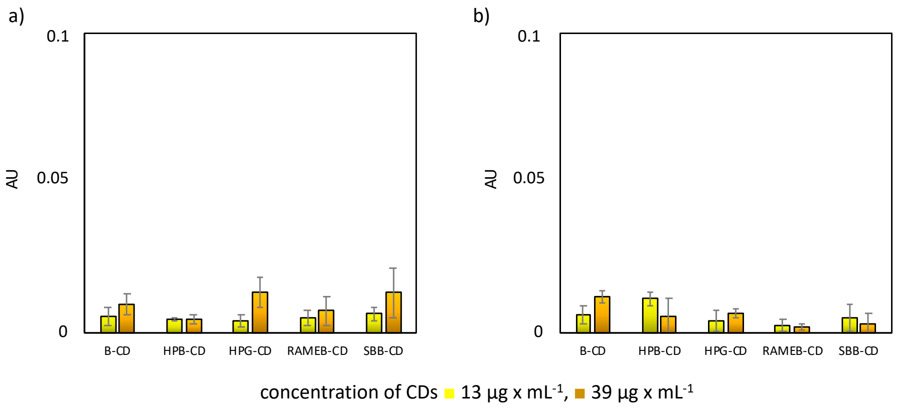

Figure S2. Investigation of the effect of CDs on the *p*-NPP hydrolysis without lipase in **a**) standard and **b**) Fessif assay, with different CD amount: 13 µg × mL^{-1} and ■ 39 µg × mL^{-1} according to the corresponding lipase:CD ratios (1:1 and 1:3 *w*/*w* respectively) in enzymatic studies (see in MS Section 2.3) by monitoring of *p*-NP formation (AU at *λ* = 400 nm for standard assay and AU at *λ* = 318 nm for FeSSIF assay) at 37.0 °C for 1 h.

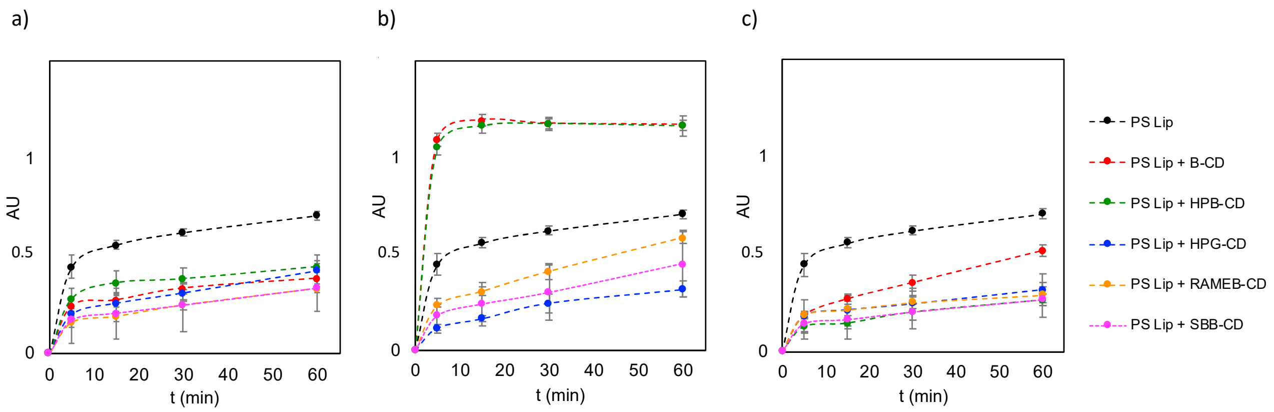

Figure S3. Effect of cyclodextrins (B-CD: β-cyclodextrin, HPB-CD: 2-hydroxypropyl-β-cyclodextrin, HPG-CD: 2-hydroxypropyl-γ-cyclodextrin, RAMEB-CD: randomly methylated β-cyclodextrin, SBB-CD: sulfobutylated-β-cyclodextrin) on the p-NP formation (AU at λ = 400 nm) from p-NPP hydrolysis catalyzed by lipase from Burkholderia cepacia (PS Lip) in standard assay. To Tris buffer (900 µL, pH = 8.0, 50 mM, 0.4% (*w*/*v*) Triton X100, 0.1% (*w*/*v*) arabic gum) a solution of p-NPP (100 µL, 16.5 mM, dissolved in 2-propanol) was added. To initiate the test reactions 150 µL of lipase solution was added [0.1 mg/mL lipase, containing CD in **a**) 1:1, **b**) 1:3 or **c**) 1:6 lipase:CD mass ratio, in Tris buffer (pH = 8.0, 50 mM, 0.4% (*w*/*v*) Triton X100, 0.1% (*w*/*v*) arabic gum)] and the resulting mixture was placed on an orbital shaker (450 rpm) at 37.0 °C.

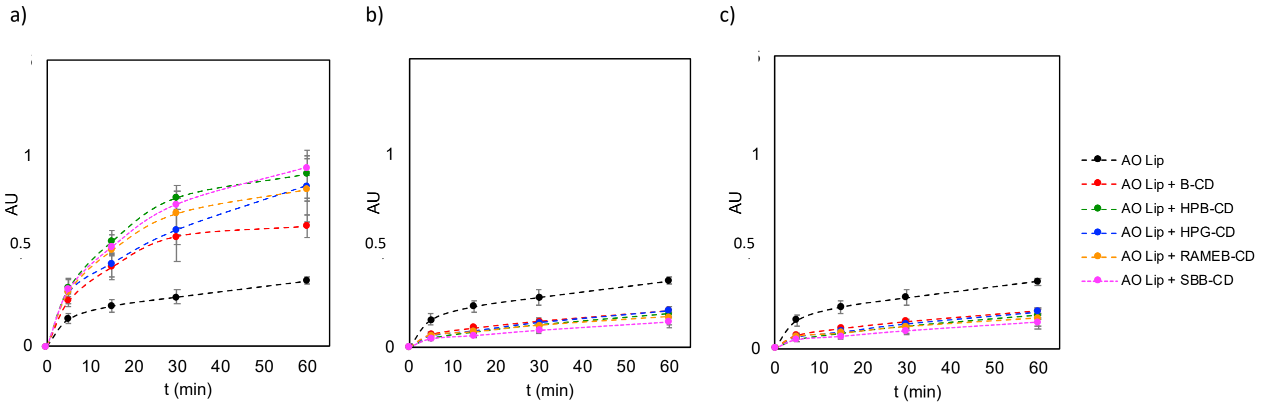

Figure S4. Effect of cyclodextrins (B-CD: β-cyclodextrin, HPB-CD: 2-hydroxypropyl-β-cyclodextrin, HPG-CD: 2-hydroxypropyl-γ-cyclodextrin, RAMEB-CD: randomly methylated β-cyclodextrin, SBB-CD: sulfobutylated-β-cyclodextrin) on the p-NP formation (AU at λ = 400 nm) from p-NPP hydrolysis catalyzed by lipase from Aspergillus oryzae (AO Lip) in standard assay. To Tris buffer (900 µL, pH = 8.0, 50 mM, 0.4% (*w*/*v*) Triton X100, 0.1% (*w*/*v*) arabic gum) a solution of p-NPP (100 µL, 16.5 mM, dissolved in 2-propanol) was added. To initiate the test reactions 150 µL of lipase solution was added [0.1 mg/mL lipase, containing CD in **a**) 1:1, **b**) 1:3 or **c**) 1:6 lipase:CD mass ratio, in Tris buffer (pH = 8.0, 50 mM, 0.4% (*w*/*v*) Triton X100, 0.1% (*w*/*v*) arabic gum)] and the resulting mixture was placed on an orbital shaker (450 rpm) at 37.0 °C.

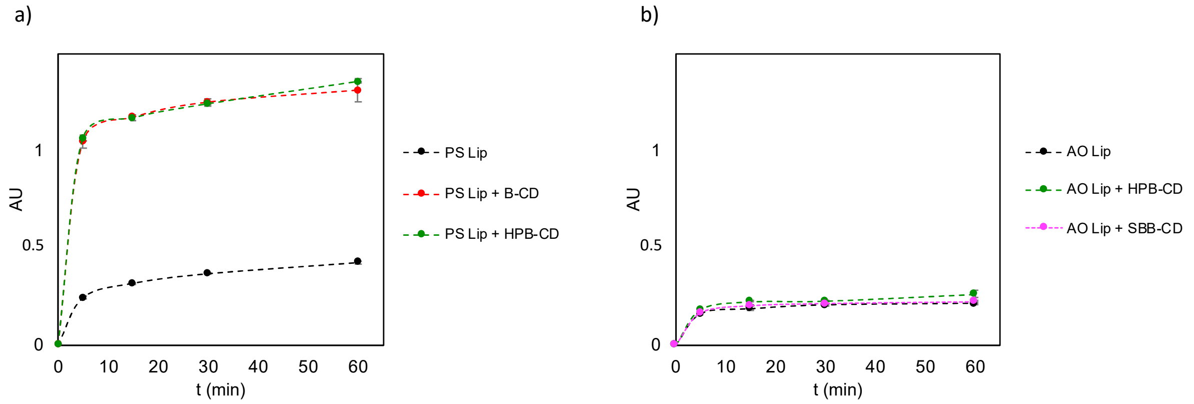

Figure S5. Effect of cyclodextrins (B-CD: β-cyclodextrin, HPB-CD: 2-hydroxypropyl-β-cyclodextrin, SBB-CD: sulfobutylated-β-cyclodextrin) on the p-NP formation (AU at λ = 318 nm) from p-NPP hydrolysis catalyzed by lipase from **a**) Burkholderia cepacia (PS Lip) and from **b**) Aspergillus oryzae (AO Lip) in FeSSIF assay. To 1.0 mL of FeSSIF solution 150 μL of lipase solution was added {0.1 mg/mL lipase, dissolved in blank FeSSIF solution, containing CD in the appropriate lipase:CD mass ratio [PS Lip:CD = 1:3, AO Lip:CD = 1:1 (*w:w*)]}, and the resulting mixture was placed on an orbital shaker (450 rpm) at 37.0 °C.

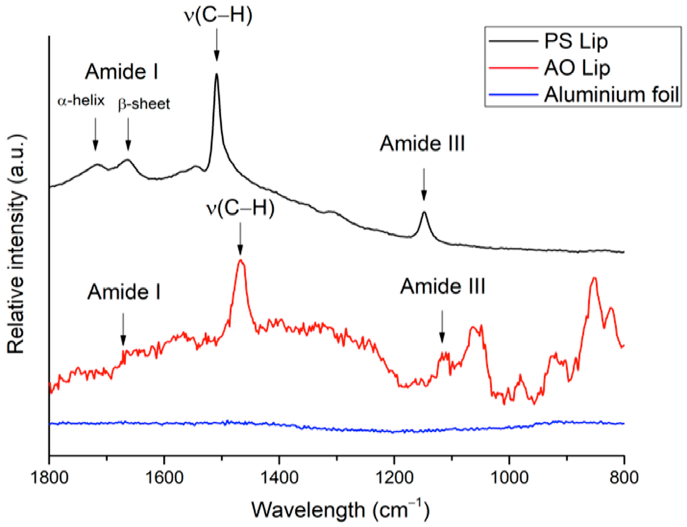

Figure S6. Raman spectra of native lipases (PS Lip: black line, AO Lip: red line).

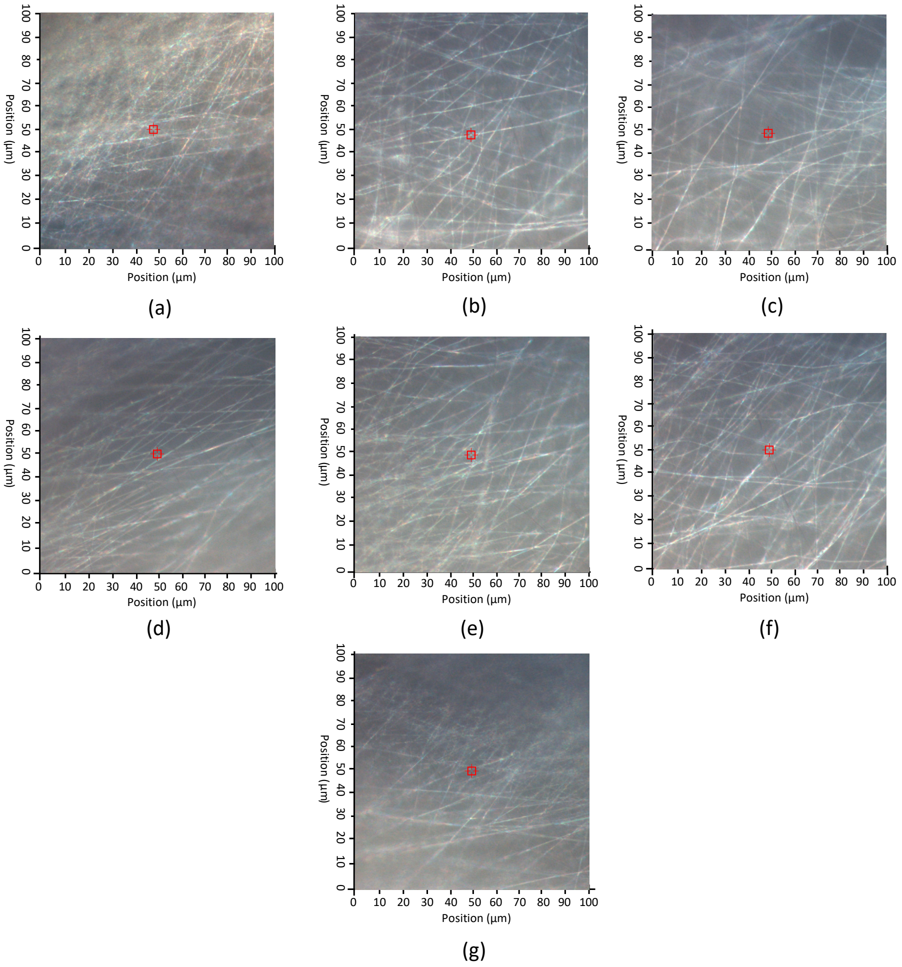

Figure S7. Mosaics of different products indicating the investigated area during Raman mapping: **a**) AO Lip-PVA, **b**) AO Lip-HP-β-CD-PVA, **c**) AO Lip-SB-β-CD-PVA, **d**) PS Lip-PVA, **e**) PS Lip-β-CD-PVA, **f**) PS Lip-HP-β-CD-PVA and **g**) initial PVA filament.

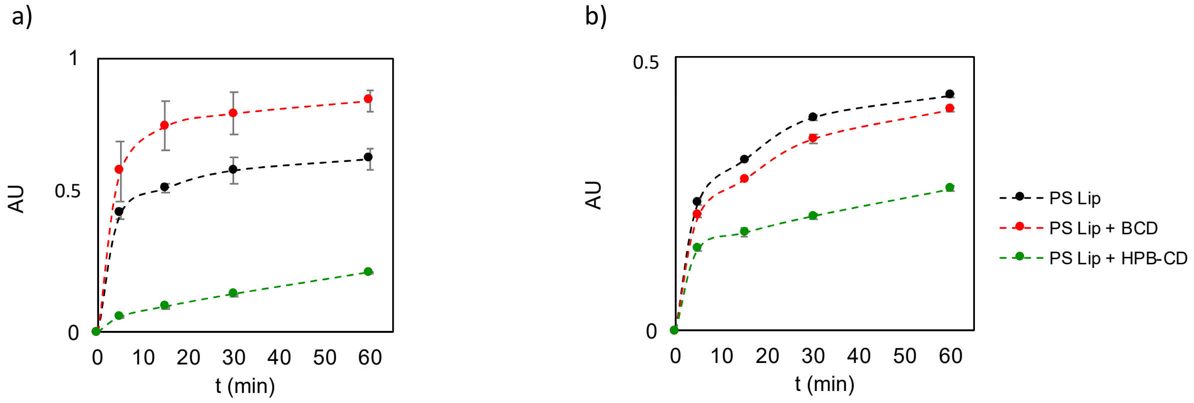

Figure S8. Effect of cyclodextrins (B-CD: β-cyclodextrin, HPB-CD: 2-hydroxypropyl-β-cyclodextrin, (PS Lip:CD = 1:3, *w:w*)) on the *p*-NP formation from *p*-NPP hydrolysis catalyzed by lipase from *Burkholderia cepacia* (PS Lip) entrapped in PVA nanofibers **a**) in standard assay (AU at λ = 400 nm) and in **b**) FeSSIF assay (AU at λ = 318 nm). Standard assay: To Tris buffer (900 µL, pH = 8.0, 50 mM, 0.4% (*w/v*) Triton X100, 0.1% (*w/v*) arabic gum) a solution of *p*-NPP (100 µL, 16.5 mM, dissolved in 2-propanol) was added. To initiate the test reactions 150 µL of lipase solution was added {5 mg nanofibrous lipase formulation, containing 10% (w/w, relative to the sum mass of the fibers) lipase and CD in the appropriate lipase:CD mass ratio, dissolved in 5.0 mL of Tris buffer (pH = 8.0, 50 mM, 0.4% (*w/v*) Triton X100, 0.1% (*w/v*) arabic gum)} and the resulting mixture was placed on an orbital shaker (450 rpm) at 37.0 °C. FeSSIF assay: To 1.0 mL of FeSSIF solution 150 µL of lipase solution was added {5 mg nanofibrous lipase formulation, containing 10% (*w/w*, relative to the sum mass of the

fibers) lipase and CD in the appropriate lipase:CD mass ratio, dissolved in 5.0 mL of blank FeSSIF solution} and the resulting mixture was placed on an orbital shaker (450 rpm) at 37.0 °C.

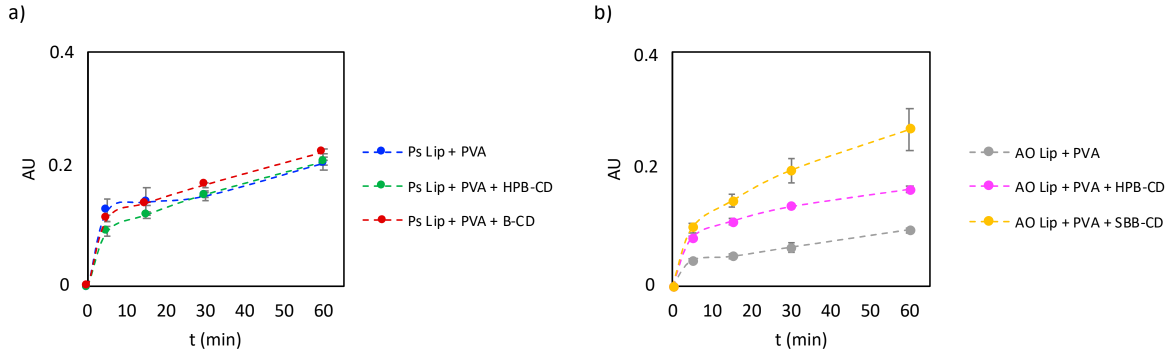

Figure S9. Effect of cyclodextrins (B-CD: β-cyclodextrin, HPB-CD: 2-hydroxypropyl-β-cyclodextrin, SBB-CD: sulfobutylated-β-cyclodextrin (AO Lip:CD = 1:1, *w:w*; PS Lip:CD = 1:3, *w/w*)) on the *p*-NP formation (AU at λ = 400 nm) from *p*-NPP hydrolysis catalyzed by **a**) lipase from *Burkholderia cepacia* (PS Lip) and **b**) lipase from *Aspergillus oryzae* in preformulation mixture containing PVA in standard assay. To carry out the tests, by dissolving Mowiol® 18-88 PVA, the appropritare lipase-CD pairs in Tris buffer, we prepared solutions with the composition corresponding to the initiator solution used in the study of the activity of the nanofibrous formulations: 5 mg of the nanofibrous formulations [containing 10% (*w/w*, relative to the sum mass of the fibers) lipase and CD in the appropriate lipase:CD mass ratio], dissolved in 5mL of Tris buffer (pH = 8.0, 50 mM, 0.4% (*w/v*) Triton X100, 0.1% (*w/v*) arabic gum). 150 µL of the resulting solutions were used to initiate the test reactions in standard assay and the resulting mixture was placed on an orbital shaker (450 rpm) at 37.0 °C. Standard assay: To Tris buffer (900 µL, pH = 8.0, 50 mM, 0.4% (*w/v*) Triton X100, 0.1% (*w/v*) arabic gum) a solution of *p*-NPP (100 µL, 16.5 mM, dissolved in 2-propanol) was added.

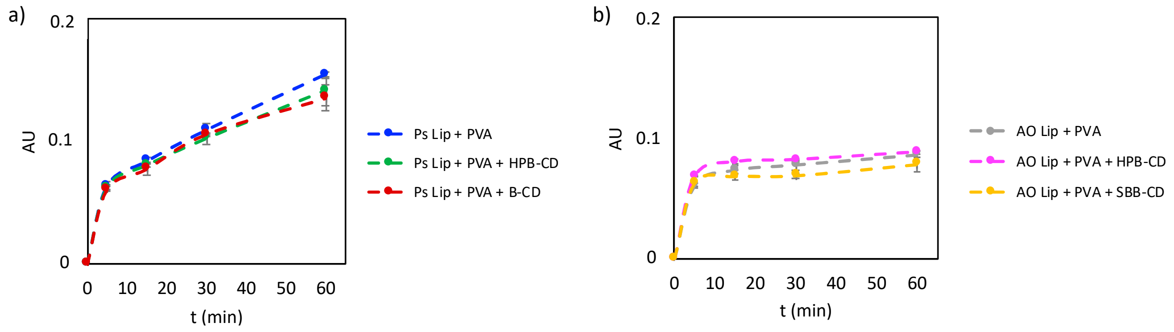

Figure S10. Effect of cyclodextrins (B-CD: β-cyclodextrin, HPB-CD: 2-hydroxypropyl-β-cyclodextrin, SBB-CD: sulfobutylated-β-cyclodextrin (AO Lip:CD = 1:1, *w:w*; PS Lip:CD = 1:3, *w/w*)) on the p-NP formation (AU at λ = 318 nm) from p-NPP hydrolysis catalyzed by **a**) lipase from Burkholderia cepacia (PS Lip) and **b**) lipase from Aspergillus oryzae in preformulation mixture containing PVA in Fessif assay. To carry out the tests, by dissolving Mowiol® 18-88 PVA and the appropritare lipase-CD pairs in blank FeSSIF, we prepared solutions with the composition corresponding to the initiator solution used in the study of the activity of the nanofibrous formulations: 5 mg of the nanofibrous formulations [containing 10% (w/w, relative to the sum mass of the fibers) lipase and CD in the appropriate lipase:CD mass ratio], dissolved in 5mL of blank FeSSIF. 150 µL of the resulting solutions were used to initiate the test reactions in 1.0 ml of FeSSIF assay and the resulting mixture was placed on an orbital shaker (450 rpm) at 37.0 °C.

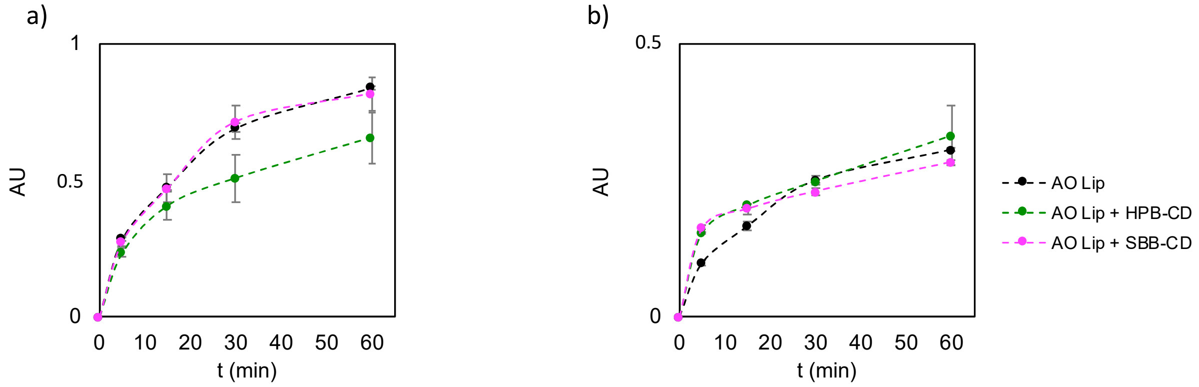

Figure S11. Effect of cyclodextrins (HPB-CD: 2-hydroxypropyl-β-cyclodextrin, SBB-CD: sulfobutylated-β-cyclodextrin (AO Lip:CD = 1:1, *w:w*)) on the p-NP formation from *p*-NPP hydrolysis catalyzed by lipase from *Aspergillus oryzae* (AO Lip) entrapped in PVA nanofibers **a**) in standard assay (AU at λ = 400 nm) and in **b**) FeSSIF assay (AU at λ = 318 nm). Standard assay: To Tris buffer (900 μL, pH = 8.0, 50 mM, 0.4% (*w/v*) Triton X100, 0.1% (*w/v*) arabic gum) a solution of *p*-NPP (100 μL, 16.5 mM, dissolved in 2-propanol) was added. To initiate the test reactions 150 μL of lipase solution was added {5 mg nanofibrous lipase formulation, containing 10% (*w/w*, relative to the sum mass of the fibers) lipase and CD in the appropriate lipase:CD mass ratio, dissolved in 5.0 mL of Tris buffer (pH = 8.0, 50 mM, 0.4% (*w/v*) Triton X100, 0.1% (*w/v*) arabic gum)} and the resulting mixture was placed on an orbital shaker (450 rpm) at 37.0 °C. FeSSIF assay: To 1.0 mL of FeSSIF solution 150 μL of lipase solution was added {5 mg nanofibrous lipase formulation, containing 10% (*w/w*, relative to the sum mass of the fibers) lipase and CD in the appropriate lipase:CD mass ratio, dissolved in 5.0 mL of blank FeSSIF solution} and the resulting mixture was placed on an orbital shaker (450 rpm) at 37.0 °C.

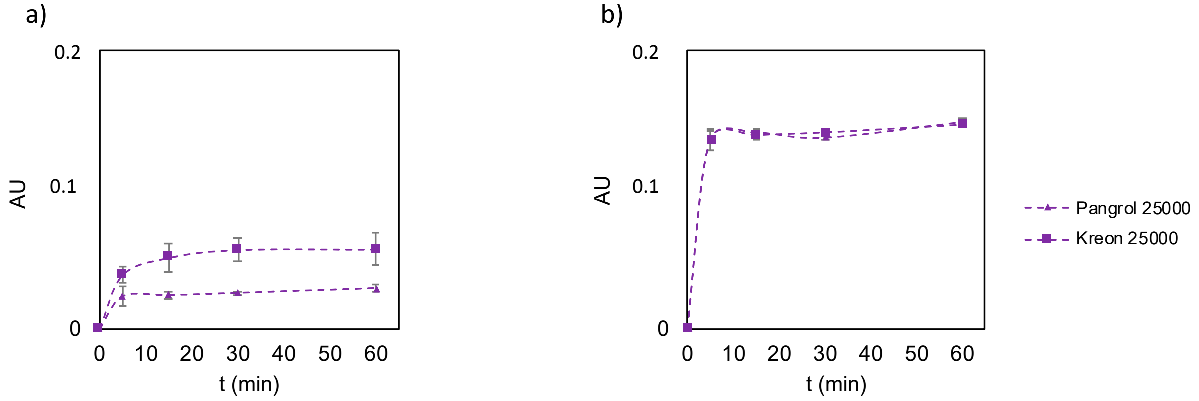

Figure S12. Investigation of commercially available medicines, Pangrol® and Kreon® by the formation of p-NP from p-NPP hydrolysis in **a**) standard assay (AU at λ = 400 nm) and **b**) FeSSIF assay (AU at λ = 318 nm) according to Section 2.9 in MS. To carry out the test reactions, one capsule was broken from each formulation, 5.0 mg of the load was dissolved in 5.0 mL of the appropriate buffer according to standard or FeSSIF assay (see sections 2.2. and 2.3. in MS), and the resulting mixtures were homogenized for 1 h using an orbital shaker (450 rpm). Subsequently 150 μL of the solutions obtained by dissolving the commercial drugs were added to 1.0 mL of FeSSIF assay and standard assay to initiate the test reactions and the resulting mixture was placed on an orbital shaker (450 rpm) at 37.0 °C. Standard assay: To Tris buffer (900 μL, pH = 8.0, 50 mM, 0.4% (*w/v*) Triton X100, 0.1% (*w/v*) arabic gum) a solution of *p*-NPP (100 μL, 16.5 mM, dissolved in 2-propanol) was added.
